# Current Tissue Molecular Markers in Colorectal Cancer: A Literature Review

**DOI:** 10.1155/2017/2605628

**Published:** 2017-10-29

**Authors:** Gaia Peluso, Paola Incollingo, Armando Calogero, Vincenzo Tammaro, Niccolò Rupealta, Gaetano Chiacchio, Maria Laura Sandoval Sotelo, Gianluca Minieri, Antonio Pisani, Eleonora Riccio, Massimo Sabbatini, Umberto Marcello Bracale, Concetta Anna Dodaro, Nicola Carlomagno

**Affiliations:** ^1^Department of Advanced Biomedical Science, University of Naples Federico II, Via S. Pansini 5, 80131 Naples, Italy; ^2^Department of Public Health, University of Naples Federico II, Via S. Pansini 5, 80131 Naples, Italy

## Abstract

**Background:**

Colorectal cancer (CRC) is one of the most spread neoplasia types all around the world, especially in western areas. It evolves from precancerous lesions and adenomatous polyps, through successive genetic and epigenetic mutations. Numerous risk factors intervene in its development and they are either environmental or genetic.

**Aim of the Review:**

Alongside common screening techniques, such as fecal screening tests, endoscopic evaluation, and CT-colonography, we have identified the most important and useful biomarkers and we have analyzed their role in the diagnosis, prevention, and prognosis of CRC.

**Conclusion:**

Biomarkers can become an important tool in the diagnostic and therapeutic process for CRC. But further studies are needed to identify a noninvasive, cost-effective, and highly sensible and specific screening test for their detection and to standardize their use in clinical practice.

## 1. Introduction

Colorectal cancer (CRC) is the third of all cancers for incidence and mortality, behind prostate and lung cancer in males and behind breast and lung cancer in females [1]. The incidence is similar in both sexes, is slightly greater in males for rectal cancer, and is higher in western countries, especially in the United States, Canada, Europe, and also New Zealand and Australia [2].

It, usually, grows in the lining of colon and rectum in the form of a polyp, a mass protruding in the lumen. Not all the polyps are neoplastic and evolve in cancer, but it is well known that the majority of colorectal cancers progress from adenomatous polyps, in the so-called adenoma-to-carcinoma sequence [3].

Mortality can be reduced through prevention and detection at an early stage; therefore, the ultimate aim should be to implement and improve the screening strategies [4, 5]. The screening techniques can be classified as noninvasive and invasive and their sensitivity and specificity are variable (Table 3). In the latest years, more attention has been paid to numerous biomarkers that could help in the early diagnosis, treatment, and prognosis of CRC. To discover these potential biomarkers, which could be detected in blood and stool through noninvasive methods, it is important to study the genetic and pathogenetic basis of CRC [6, 7].

## 2. Risk Factors

In the development of CRC environmental and genetic factors play a very important role, Tables 1 and 2.

## 3. Biomarkers

A molecular marker or biomarker is a molecule able to be detected in tissue or serum and that allows identifying a particular condition or a disease. Biomarkers have a high prognostic and predictive value and are an important instrument for the early diagnosis of CRC, for its treatment, and for the patients' outcome [52].

These markers can be divided into three different groups: diagnostic, predictive, and prognostic.

Diagnostic markers permit an early diagnosis and risk stratification.

Predictive biomarkers are useful for predicting the patient's response to the therapy and so patients can be selected to undergo a particular treatment on the basis of a likely positive response. They can even be used to identify the right drug dose and to prevent its toxicity [53–56].

Prognostic biomarkers allow estimating the natural course of the disease and dividing tumors in two groups: the ones with a good outcome and the ones with a bad outcome [57]. They can be molecules involved in different process, such as cellular proliferation, differentiation, angiogenesis, invasion, and metastasis [53].

Mutations of KRAS, BRAF, and MSI are the ones most commonly detected during the diagnostic and therapeutic process of CRC to better define the most proper treatment.

### 3.1. Diagnostic

#### 3.1.1. Microsatellite Instability (MSI)

Microsatellites are short sequences of 1–6 base pairs in the genome that have a major risk of mutations which are corrected by the MMR systems. HNPCC is caused by a germ line mutation of one of the four MMR genes, MSH2, MLH1, MSH6, and PMS2, that leads to Microsatellites Instability (MSI) [37]. MSI is also responsible for sporadic CRC. Five MS markers have been identified: 2 mononucleotides (BAT 25 and BAT 26) and 3 dinucleotides (D2S123, D5S346, and D17S250). These are sought in tissues when HNPCC is suspected and, if positive, in the serum of other family members [52, 58].

It is reported in the literature that MSI has a higher prevalence in stage II CRC and that cancers with MSI have a better prognosis than the ones characterized by microsatellite stability. Therefore, MSI can be not only a diagnostic tool but even a useful prognostic factor [59].

#### 3.1.2. Insulin-Like Growth Factor Binding Protein 2 (IGFBP2)

IGFBP-2 is a protein that modulates the binding between IGF and IGF-1. In CRC its levels are increased for an overexpression of its mRNA [60].

Serum and plasma levels of IGFBP-2 are significantly higher in CRC patients than in controls and in patients with advanced tumors compared to the ones at early stages [61].

#### 3.1.3. Telomerase

Telomeres are specialized terminal structures in eukaryotic chromosomes that consist in repeats of a DNA sequence (TTAGGG), whose length is maintained by the enzyme Telomerase. Numerous studies have demonstrated an increased Telomerase Activity (TA) in CRC samples compared to normal colorectal mucosa. Some authors have also found that TA and telomeres length are independent prognostic elements to predict recurrence and disease-free and overall prognosis [62, 63].

#### 3.1.4. Pyruvate Kinase M2 (PKM2)

Pyruvate Kinase M2 is a glycolytic enzyme that plays an important role in cellular metabolism of many types of tumors. It can be detected even in normal colic cells, but its level is higher in CRC cells. Mutated PKM2 can be detected in stool, with ELISA technique, but its role as diagnostic marker must be further studied [64, 65].

### 3.2. Predictive

#### 3.2.1. KRAS

Mutation of KRAS is of the most common alterations in CRC. The majority of these mutations happen in codons 12 and 13 and are DNA base pair substitutions with subsequent amino acid changes in the protein [66]. They cause an activation of EGFR pathway which becomes independent from EGFR activation. It is reported that these mutations are associated with chemoresistance to Anti-EGFR Antibodies, Cetuximab, and Panitumumab. Thus mutated KRAS is the most important predictive factor of the response to EGFR inhibitors [67, 68].

According to some authors KRAS mutation is also related to a poor prognosis, whereas in other studies it is shown that it has no major prognostic value [66, 69–72].

Recent studies have reported that even mutations of NRAS, which occur in 3–5% of CRC, determine a negative response to anti-EGFR therapy [67].

#### 3.2.2. BRAF

BRAF is frequently mutated in CRC; the most common mutation is V600E that leads to a glutamic acid for valine substitution in the protein, causing the constitutional activation of MAPK pathway. BRAF and RAS mutations are, usually, mutually exclusive. BRAF V600E mutation is sought for two reasons: in MSI CRC can exclude Lynch Syndrome and in the MSS (microsatellite stable) ones is associated with a poor prognosis. It determines, in fact, as well as RAS mutation, resistance to the anti-EGFR therapy. Traditionally it has been detected with a PCR analysis, while recently immunohistochemistry has been approved for its research [73–75].

#### 3.2.3. PIK3CA

Phosphoinositide-3-kinase is an enzyme of the AKT pathway. Its alteration determines an activation of the pathway and cell proliferation. Mutation in exon 20 is significantly associated with a low response to treatment with the monoclonal antibody anti-EGFR Cetuximab and with a worse prognosis if compared to patients with wild-type PIK3 [67, 76].

#### 3.2.4. PTEN (Phosphatase and Tensin Homolog Protein)

PTEN is a tumor suppressor gene, whose inactivation causes deregulation of the PI3K pathway. The loss of PTEN has been associated with aggressive CRCs and is predictive of a nonresponse to the treatment with Cetuximab [77, 78].

It is also a predictive factor for tumor with wild-type KRAS treated with anti-EGFR therapy [77, 79].

#### 3.2.5. ERCC-1

Excision repair cross-complementing-1 is part of a family of genes that prevent DNA damage by nucleotide excision and repair. Level of its mRNA in cancer cells correlates with response to the therapy with oxaliplatin. Patients with low level show a better outcome than the ones with a higher number of copies of its mRNA; it has been hypothesized that an increased DNA repair antagonized the effect of platinum-based treatments [80].

#### 3.2.6. Ezrin

Ezrin is a cytoskeletal protein that plays an important role in cell motility, invasion, and metastasis. Hyperphosphorylation at the site T567 has been sought in liver metastases, but its levels were lower in the primary tumor [81]. An increased cytoplasmatic expression of Ezrin correlates with a greater aggressiveness of CRC and therefore with a poor prognosis. Ezrin could become a target for antimetastatic therapy. Two small molecules, NSC305787 and NSC668394, which bind Ezrin and prevent its phosphorylation and activation, are currently under study [82, 83].

#### 3.2.7. Cyclooxygenase-2

Cox-2 is involved in colorectal carcinogenesis. Its level is increased in the majority of CRCs, especially in advanced stages. It could have an important role as prognostic and predictive factor [6].

### 3.3. Prognostic

#### 3.3.1. APC

Adenomatous Polyposis Coli is an oncosuppressor gene, whose mutation in germ line is responsible for FAP, but it is also mutated in the majority of sporadic CRCs. Even hypermethylation of APC gene promoter has been implicated in the development of colorectal adenomas and cancers [84, 85]. Both of these mechanisms lead to APC inactivation and this is considered a poor prognostic factor [86].

#### 3.3.2. p53

TP53 gene mutation is one of the hallmarks of human tumors and plays an important role in the development of CRC [80]. Numerous studies have reported how its dysfunctions, more often caused by missense mutations, can be used as prognostic markers. It has been demonstrated that in almost half of the patients' serum antibodies anti-p53 can be detected, but the role in tumor screening must be further investigated [87, 88].

#### 3.3.3. VEGF

Vascular endothelial growth factor is an angiogenetic factor involved in CRC and indirectly responsible for tumor growth and metastases. Its mutations are associated with a greater aggressiveness and poor prognosis and can be at the basis of resistance to anti-EGFR treatment [52, 89].

#### 3.3.4. EGFR (Epidermal Growth Factor Receptor)

EGFR is a transmembrane tyrosine kinase receptor, which is overexpressed in various tumors, including CRC. Two monoclonal antibodies, Cetuximab and Panitumumab, are currently used in treatment of CRCs presenting this overexpression, as monotherapy or in combined chemotherapy [77, 90, 91].

#### 3.3.5. 18q Loss of Heterozygosity (LOH)

Allelic loss of chromosome 18q is observed in up to 70% of CRCs and is associated with a poorer prognosis. Patients with stage II or III cancer that present LOH are shown to have a worse outcome compared to the ones with both allelic copies and could benefit from an adjuvant chemotherapy [92, 93].

#### 3.3.6. SMAD4

SMAD4 is an oncosuppressor protein that intervenes in the intracellular pathway of TGF-*β*. Its inactivation leads to altered TGF-*β* signaling and is related to tumor invasion, metastases formation, and poor response to chemotherapy. Thus, SMAD4 is a valuable prognostic marker [94, 95].

#### 3.3.7. Mutated in Colorectal Cancer (MCC)

MCC is a multifunctional protein that enters in the Wnt and NFkB pathways [96]. Mutations or loss of heterozygosity of its gene, located on chromosome 5q21, has been associated with CRC. MMC binds *β*-catenin, hindering the Wnt/*β*-catenin signaling pathway, and so it could have a prognostic value [97].

#### 3.3.8. Insulin-Like Growth Factor II mRNA-Binding Protein 3 (IMP3)

IMP3 is a protein expressed during embryogenesis and is almost undetectable in adult tissues but is expressed in neoplastic cells. It is reported that its expression in CRC is related to a more aggressive phenotype. It is considered an important prognostic marker and a predictor for metastases' formation [98].

#### 3.3.9. TRAF2- and NICK-Interactive Kinase (TNIK)

TNIK is a kinase involved in cytoskeleton organization and neural dendrite extension and is activated by the binding with *β*-catenin. High levels of TNIK are present in CRC and they are related to distant metastases in stage II and III tumors [99].

#### 3.3.10. S100A2 Protein

S100 calcium-binding protein A2 (S100A2), a protein involved in cell cycle progression, has been demonstrated to be implicated in the distant metastasis of stage II and III CRC. Thus it can be used as a marker for the recurrence's prediction [100].

## 4. Conclusion

Biomarkers can be an important tool for early detection and prevention of CRC and guide the therapeutic process with a personalized therapy, on the basis of the presence of defined markers. Nowadays, there is not still a universal biomarker of CRC that allows a satisfying secondary prevention of this disease. Thus it is important to continue the study of the genetic and epigenetic modifications that underlie the CRC to discover new biomarkers. The main aim of future researches should be to perfect a noninvasive, cost-effective screening test with a high sensitivity and specificity that will allow the detection of a panel of biomarkers that can be employed in the clinical practice. Only through wide prospective studies on large series, it will be possible to validate the emerging biomarkers and standardize their practical use.

## Figures and Tables

**Table 1:** Environmental risk factors.

Environmental factors	
Age	The risk of developing CRC increases with age and the majority of the cases are diagnosed in patients older than 50 years [8–10]. A higher prevalence is reported in people aged over 60 years compared to those younger than 40 years [2].

Gender	In the literature the incidence of CRC is the same in males and females. Females are shown to be older and to have right-sided tumors and less advanced diseases [11].

Westernized lifestyle	Long-term smoking is strongly associated with the development of adenomatous polyps and is important for both formation and aggressiveness [12]. Recent meta-analyses point out a statistically significant increase of risk after 30 years of smoking, especially in CRCs displaying MSI. A greater association with rectal and proximal colon tumors is also reported [13, 14].
	Diet is surely one of the most important risk factors, especially one rich in red meat. This association between red meat and cancer, stronger for the colon cancer, may depend on the presence of heme iron in meat [15–17].
	Alcohol consumption also is a known risk factor for CRC. The interference on the folate synthesis, with the production of acetaldehyde that degrades folate, may be at the basis of the chromosome damage and so of the carcinogenesis process [18, 19].

**Table 2:** Genetic risk factors.

Genetic factors	
APC	The Adenomatous Polyposis Coli (APC) gene, located on chromosome 5, is a tumor suppressor, which is mutated in most of sporadic cases of colon adenocarcinomas. APC mutation leads to an increased amount of *β*-catenin and to the activation of the Wnt signaling pathway that is involved in cellular activation [20–22].

Chromosomal instability	Chromosomal instability is a common factor that intervenes in the adenoma-carcinoma sequence. It causes the inactivation of wild-type allele of tumor suppressor genes, such as SMAD4, APC, and p53, the loss of heterozygosity, and the alteration in chromosome number, like aneuploidy [22–24].

BRAF and RAS	RAS and RAF are two oncogenes which activate the mitogen-activated protein kinase (MAPK) pathway. KRAS has a GTPase activity that activates RAF proteins; BRAF's serine-threonine kinase activity initiates the MAPK signaling cascade, with the activation of several transcription factors. The result is cell survival, proliferation, and metastasis [25]. Already small polyps present BRAF mutation, whereas in serrated adenomas, hyperplastic polyps and proximal colon cancer RAS is more often mutated [26, 27].

DCC	Deleted in Colorectal Cancer (DCC) is a tumor suppressor gene sited on the long arm of chromosome 18 (18q21.3). It is a transmembrane protein that stops cell growth in absence of Netrin and its ligand. Its mutation prevents the bond with Netrin-1 and results in abnormal cell survival. Loss of heterozygosity (LOH) of chromosome 18q is seen in more than 70% of advanced CRC [23, 28, 29].

Family history	FAP, Familiar Adenomatous Polyposis, is an autosomal dominant disease caused by germ line mutation of APC gene. Patients affected by FAP develop thousands of polyps in gastrointestinal system, especially in the colon, starting from the second decade of life; if not treated they will develop a CRC in early adulthood [30–35].
	Hereditary nonpolyposis colorectal cancer (HNPCC) or Lynch Syndrome is the most common hereditary form of CRC (2–4% of all CRC) [30, 36]. A characteristic trait of NHPCC is Microsatellite Instability (MIS) due to the inherited mutation of the Mismatch Repair Genes (MMR) that control the length of microsatellites, short nucleotides' sequences repeated in DNA [37, 38].

**Table 3:** Current screening options.

Screening options	
Fecal screening tests	These tests search for occult blood in stool, which is nonspecific but can be detected especially in larger polyps and CRC. It is important to collect samples from consecutive bowel movements [39, 40]. Guaiac fecal occult blood test (gFOBT) detects qualitatively heme in the stool, using a guaiac material to which hydroperoxidase is added. Heme promotes a process that leads to the guaiac's oxygenation and to a blue discoloration [41, 42]. It has a low sensitivity for the detection of CRC, but when performed every year or two, mortality is reduced [39, 43, 44]. Fecal immunochemical tests (FITs) use monoclonal or polyclonal antibodies to detect human haemoglobin. They can give qualitative or quantitative results. FIT is more accurate than gFOBT, because it does not react with nonhuman heme and is less sensitive to upper gastrointestinal tract's bleeding [41, 45].

Endoscopic screening	Flexible Sigmoidoscopy is a screening option that allows examining the rectum and the lower part of the colon. It is an invasive technique that requires simple bowel preparation but cannot detect lesion in the whole colon [41, 45]. Colonoscopy is esteemed as the gold standard for CRC screening; it allows exploring the whole colon and removing the suspicious lesions [46, 47]. It is an invasive and expensive exam that must be performed if any other test has a positive result [39]. The most common side effect is postpolypectomy bleeding, but also tearing and perforation may be possible [45]. It is recommended that colonoscopy be practiced every 10 years in average-risk patients that underwent to a complete, negative exam [39, 48, 49].

CT-colonography (CTC)	CTC is a noninvasive test that has become a common method for CRC screening. It requires a bowel preparation, but sedation is not needed. The estimated sensitivity and sensibility in detecting polyps > 1 cm are high, above 90%. Limitation of this technique includes low sensitivity for small lesion and serrated polyps, the exposure to radiation, and the need of follow-up for extra colic incidental findings [39, 49–51].
